# Transcriptome Analysis of Rat Lungs Exposed to Moxa Smoke after Acute Toxicity Testing

**DOI:** 10.1155/2021/5107441

**Published:** 2021-12-18

**Authors:** Xiaoyu Xu, Wen Deng, Wanqing Zhang, Junhua Zhang, Muchen Wang, Si Shan, Hongning Liu

**Affiliations:** ^1^Jiangxi Province Key Laboratory of TCM Etiopathogenesis, Jiangxi University of Chinese Medicine, Nanchang, Jiangxi 330004, China; ^2^Research Center for Differentiation and Development of TCM Basic Theory, Jiangxi University of Chinese Medicine, Nanchang, Jiangxi 330004, China

## Abstract

The increasing use of moxibustion has led to a debate concerning the safety of this treatment in human patients. Inhalation of cigarette smoke induces lung inflammation and granulomas, the proliferation of alveolar epithelial cells, and other toxic effects; therefore, it is important to assess the influence of inhaled moxa smoke on the lungs. In the present study, a novel poisoning cabinet was designed and used to assess the acute toxicity of moxa smoke in rats. We evaluated pathological changes in rat lung tissue and analyzed differentially expressed genes (DEGs) using RNA-seq and transcriptomic analyses. Our results show that the maximum tolerable dose of moxa smoke was 290.036 g/m³ and LC_50_ was 537.65 g/m³. Compared with that of the control group, the degree of inflammatory cell infiltration in the lung tissues of group A rats (all dead group) was increased, while that in group E rats (all live group) remained unchanged. GO and KEGG enrichment analyses showed that the DEGs implicated in cell components, binding, and cancer were significantly enriched in the experimental groups compared with the profile of the control group. The expressions of *MAFF*, *HSPA1B*, *HSPA1A*, *AOC1*, and *MX2* determined using quantitative real-time PCR were similar to those determined using RNA-seq, confirming the reliability of RNA-seq data. Overall, our results provide a basis for future evaluations of moxibustion safety and the development of moxibustion-based technology.

## 1. Introduction

Moxibustion and acupuncture are important practices in traditional Chinese medicine (TCM). Decades ago, moxibustion was used as mainstream therapy in TCM; based on the famous TCM text entitled Yellow Emperors Internal Classic, moxibustion was utilized in cases where other medicinal approaches and acupuncture needles were ineffective [1]. Today, moxibustion is widely used in Japan, South Korea, North Korea, Malaysia, and other countries because of its straightforward procedure and predictable curative effect [2]. However, the aromatic-smelling moxa smoke produced during the moxibustion process contains inhalable particles that may adversely affect human health [3, 4], leading to questions regarding the safety of this therapeutic technique.

Currently, few studies have evaluated the environmental toxicity of moxa smoke and air quality standards in the moxibustion environment. In our present study, we tested the acute toxicity of moxa smoke to provide a dose reference for the design of an experimental method that can be used to analyze subacute, subchronic, and chronic toxicity. To perform an acute toxicity test using respiratory tract inhalation, experimental animals are first placed in a poisoning cabinet having fixed volume. Then, specific volumes of the test substance (gas or volatile liquid) are introduced into the cabinet in order to attain targeted air concentration [5]. After exposure (usually lasting 2 h), the toxic reaction of the animals is recorded, and LC_50_ of the test substance is calculated based on the concentration of the substance and the number of dead animals. According to Neijing's “Suwen Wuzang generation,” all Qi belongs to the lung, meaning that the lung governs the Qi, which, in turn, governs breathing and the whole body [6]. Because particles in moxa smoke are small enough to penetrate the protective filters of the respiratory system and form deposits in the lungs, it is important to evaluate the effect of these particles, at different smoke concentrations, on the lung.

High-throughput sequencing has revolutionized genomic research because of its low cost and ultrahigh data output. RNA sequencing (RNA-seq) is a high-throughput method used to determine transcript abundance and identify novel transcriptionally active regions [7–9]. In lung-injury research, RNA-seq is used to effectively detect differentially expressed genes and their mechanisms. For example, Piyadasa et al. used RNA-seq to characterize the immunological and physiological responses induced by intranasal IL-33 challenge in a mouse model. Their results showed that 2,279 transcripts in lung tissues are upregulated and 1,378 are downregulated (2-fold, *P* < 0.01) upon exposure to IL-33. Moreover, the mRNA and protein expression levels of STAT4, a predicted upstream regulator of IL-33-induced transcripts, are significantly enhanced in lungs following the IL-33 challenge [10]. Currently, few RNA-seq studies have examined the mechanisms mediating moxa smoke exposure-induced lung injury in rats; to the best of our knowledge, our present study is the first to investigate these mechanisms using acute toxicity assessment. Herein, we used transcriptomic analysis to investigate these mechanisms in the lung after acute toxicity assessment. The results obtained in our present study can be used to evaluate the safety of moxibustion as a therapeutic technique.

## 2. Materials and Methods

### 2.1. Animals

Thirty-six 8-week-old Sprague Dawley male rats were obtained from Slake Jingda Experimental Animal Co., Ltd., Hunan, China (license No. of the animal: SYXK-2019-0004; license No. of the experimental unit: SYXK-2017-0004). The rats were housed in EVC cages at a controlled temperature (TEMP) of 22–25°C and relative humidity (RH) of 30–70% using a 12-h light-dark cycle. Food and water were provided ad libitum. All procedures involving animals were approved by the Ethics Committee on Animal Research of the Jiangxi University of Traditional Chinese Medicine in Nanchang, China. All experimental procedures involving animals were conducted in accordance with the International Guiding Principles for Biomedical Research Involving Animals issued by the World Health Organization.

### 2.2. Acute Poisoning Cabinet

**Figure 1:**
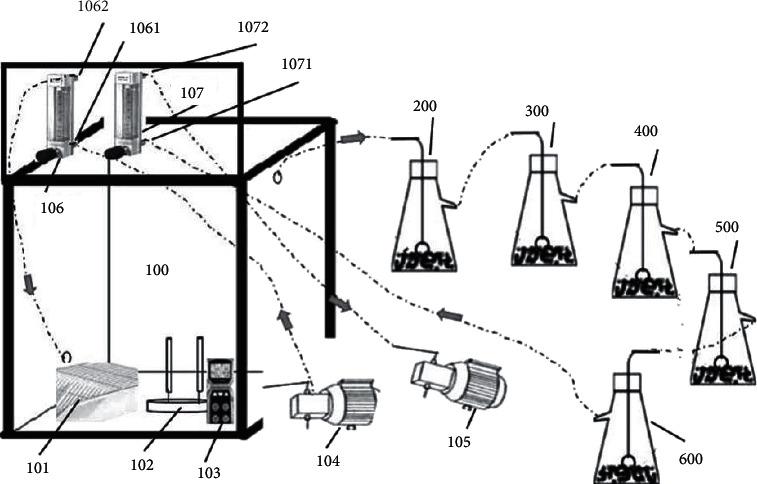
Emergency poisoning cabinet constructed in-house.

As shown in Figure 1, the 500 mm × 500 mm × 800 mm poisoning cabinet (Patent No. ZL 2020 2 0634748.8) was constructed in-house using plexiglass, which is a material characterized by high transparency and chemical stability, favorable mechanical properties, and strong resistance to weather-related stresses. A squirrel cage (101), a moxa smoke combustion fixing frame (102), and an O_2_ detector (103) were incorporated into the moxa smoke bin (100). An air inlet pump (104) and a flow meter (106) were affixed to the lower right side of the cabinet in order to introduce air into the poisoning cabinet. The inlet (1061) and outlet (1062) of the flow meter (106) were directly connected to the inlet pump (104) and moxa smoke bin (100), respectively. A second pump (105) and flow meter (107), placed at the upper left side of the cabinet, were used to sample the air inside the poisoning cabinet. The inlet (1071) and outlet (1072) of the outlet flow meter were directly connected to the outlet of the moxa smoke absorption device and the air outlet pump (105), respectively. The air inlet of the moxa smoke absorption device, composed of cyclohexane (200), ethyl acetate (300), n-butanol (400), anhydrous ethanol (500), and water (600) solvent traps having increasing polarity, was connected to the air outlet on the exposure cabinet.

### 2.3. Measurement of Basic Combustion Parameters of Moxa Sticks

#### 2.3.1. Measurement of Moisture Content

To measure the moisture content in moxa sticks, we first recorded the initial mass (m0) of moxa sticks. Then, moxa sticks were dried in an oven at 103 ± 2°C to constant weight. After recording the mass of the dried sticks (m1), the absolute (W_1_) and relative (W_2_) moisture contents were calculated as follows:

(1) $$\begin{array}{c} W_{1}=\frac{m0-m1}{m1}\mathrm{\times 100}\%,\\ W_{2}=\frac{m0-m1}{m0}\mathrm{\times 100}\%. \end{array}$$

#### 2.3.2. Measurement of Ash Content

Preweighed moxa sticks (initial mass A1) were placed on a combustion rack; after undergoing combustion, the mass of the moxa sticks was measured again (A2). The absolute (S_1_) and relative (S_2_) ash contents were calculated according to the following formulas:

(2) $$\begin{array}{c} S_{1}=\frac{A2}{A1\times \;\left(1-W2\right)\;}\times 100\%,\\ S_{2}=\frac{A2}{A1}\times 100\%. \end{array}$$

#### 2.3.3. Measurement of Smoke Generation Rate

According to the law of mass conservation, the rate of smoke generation is equivalent to the collective amount of all substances in the combustion space except for moisture, ash, and oxygen consumed during combustion [11]. To determine the smoke generation rate of moxa sticks, a certain mass of sticks having known moisture content was introduced into the cabinet and combusted in an atmosphere of known oxygen concentration. After combustion, the produced ash was weighed, and the concentration of remaining oxygen was determined. Finally, the generation rate of moxa smoke was calculated based on the following formula:

(3) $$\begin{array}{c} M_{1}=M_{2}-M_{3}-M_{4}+M_{5},\\ =M_{2}-M_{2}\times W_{2}-M_{4}+\rho \left(C_{1}-C_{2}\right)V,\\ =M_{2}\left(1-W_{2}\right)-M_{4}+\rho \left(C_{1}-C_{2}\right)V, \end{array}$$

where *M*_2_ (*g*) and *M*_4_ (*g*) are the masses of moxa sticks before and after burning, respectively; *ρ* is the density of O_2_ (1.429 g/L); *C*_1_ (%) and *C*_2_ (%) are the concentrations of oxygen in the cabinet before and after burning, respectively; and *V* is the volume of the chamber (200 L).

### 2.4. Exposure to Moxa Smoke

In our present study, we investigated the toxicity profile of Qing moxa sticks (18 mm × 200 mm × 10) purchased from Jiangxi Poai Biotechnology Co., Ltd. (Poyang, China); the moxa sticks were encased in *Artemisia argyi* (Chinese mugwort) floss made of dried *A. argyi* leaves at the ratio of 10 : 1 (i.e., 10 kg of dried *A. argyi* leaves encased 1 kg of moxa sticks). Before burning, these sticks were conditioned in a sealed room at 20–25°C for 7–21 days.

To study the acute toxicity of moxa smoke in the context of respiratory inhalation, the rats were randomly divided into five experimental groups (A, B, C, D, and E) with six rats per group. The rats were exposed to moxa smoke for 2 hours per day, for 14 consecutive days. To avoid death by asphyxiation during exposure to moxa smoke, the concentration of oxygen in the cabinet was maintained at 19–20 vol% using constant air flow at the rate of 30 L/min. The mental state, activity, and condition of the rats were closely observed during the experiment. On day 14 (which concluded this experiment), the lungs of the rats were removed and weighed in order to determine the organ index (organ weight/body weight). Histopathological changes in lung tissue were observed by hematoxylin and eosin staining (H&E).

### 2.5. Calculation of LC50

The median lethal concentration (LC_50_) of moxa smoke was determined using Karber's method based on the results of the acute toxicity test. The following formula was used:

(4) $$\begin{array}{c} L{C\text{LC}}_{50}={\mathrm{lg}}^{-1}\left[X_{m}-i\left(\sum P-0.5\right)\right], \end{array}$$

where *X*_*m*_ is the logarithm of maximum dose, *i* is the logarithm of the ratio of two adjacent groups (lgr), *P* is mortality in each group, and ∑ *P* is total mortality.

### 2.6. Microscopic Examination

After acute toxicity testing, each rat was subjected to full necropsy. The middle lobe of the right lung was collected, preserved in 10% neutral buffered formalin, desiccated, and embedded in paraffin. Five-micrometer-thick sections of the dissected lung samples were stained using H&E for histopathological observation under a light microscope (Nikon).

### 2.7. RNA Extraction and Construction of cDNA Library

Total RNA was extracted from the lung tissues of control rats and those belonging to groups A, B, and E using Trizol reagent (Invitrogen) according to the manufacturer's instructions. Genomic DNA was removed using DNase I (Takara). RNA quality was determined using a 2100 Bioanalyzer (Agilent). ND-2000 (NanoDrop Technologies) was used to quantify the extracted RNA. Only high-quality RNA samples (OD260/280 = 1.8–2.2, OD260/230 ≥ 2.0, RIN ≥ 6.5, 28S : 18S ≥ 1.0, >2 *μ*g) were used to construct the sequencing library.

The RNA-seq transcriptome library was prepared using 1 *μ*g total RNA and a TruSeq^TM^ RNA sample preparation kit from Illumina (San Diego, CA). Messenger RNA (mRNA) was isolated using poly-(A) selection with oligo (dT) beads. Then, mRNA was fragmented using a fragmentation buffer. Subsequently, double-stranded cDNA was synthesized using a SuperScript double-stranded cDNA synthesis kit (Invitrogen, CA) with random hexamer primers (Illumina). The synthesized cDNA was subjected to end-repair, phosphorylation, and “A” base addition according to the Illumina library construction protocol. Adapter-modified fragments were selected using gel purification and PCR amplification and were then used to create the final cDNA library.

### 2.8. Transcriptome Sequencing and Bioinformatics Analysis

Transcriptome sequencing was performed using an Illumina HiSeq 4000 high-throughput sequencing platform (Illumina, San Diego, CA, USA). The original sequencing images of 5 *μ*g total RNA were converted to sequential data using base calling. Raw paired-end reads were trimmed and quality controlled using SeqPrep (https://github.com/jstjohn/SeqPrep) and Sickle (https://github.com/najoshi/sickle) at default parameters. Subsequently, clean reads were separately aligned with the reference genome using TopHat software version 2.0.0 (http://tophat.cbcb.umd.edu/) in orientation mode [12]. The human genome sequence and gene annotation were obtained from the UCSC Genome Website, and Cuffdiff was used to profile the differentially expressed genes at default parameters [13]. To identify homologs (*E*-value < 10^−10^), unigenes were queried against GO, KEGG, COG, NR, Swiss-Prot, and Pfam databases using BLASTx [14–19]. The differentially expressed genes (DEGs) in groups A (all dead) and E (all living) and those in the control group (no exposure to moxa smoke) were identified and compared based on fragments per kilobases per million reads (FPKM) using RSEM 1.2.31 software [20]. DESeq was used to determine the false discovery rate (FDR) threshold (adjusted *P* value) [21]. In multigroup comparisons, FDR values less than 0.05 indicate significantly different expression levels.

### 2.9. Quantitative RT-PCR (qRT-PCR) Analysis of Gene Expression

Total RNA extracted from the lung tissues of rats in groups A, B, and E and that of control rats were analyzed using qRT-PCR. Briefly, 2 *μ*g each total RNA sample was reverse transcribed and quantified using an SYBR Green real-time PCR Master Mix kit (Takara, Dalian, China). The relative expression of each gene was calculated and normalized relative to that of *GAPDH*, used as endogenous control, according to the 2^−∆∆Ct^ method. Assays were performed in quadruplicate.

### 2.10. Statistical Analysis

Numerical data are expressed as mean ± standard deviation (SD). Differences between means were analyzed using SPSS 21.0 software (SPSS Inc., Chicago, IL, USA). A two-tailed Student's *t*-test was used to compare differences between the groups, and *P* values less than 0.05 (^*∗*^) or 0.01 (^∗∗^) were considered statistically significant.

## 3. Results

### 3.1. Moisture and Ash Content and Rate of Smoke Regeneration

As shown in Table 1, the average relative and absolute moisture contents in Qing moxa sticks were 9.59 and 10.62%, respectively. Because these contents were not excessively high, the sticks were not prone to mildew during storage. The amount of ash produced upon the burning of moxa sticks was relatively small (Table 2), indicating that the sticks underwent complete combustion during which most of their components volatilized. On average, the absolute and relative ash contents of moxa sticks were 7.88 and 7.12%, respectively. According to the law of mass conservation, the rate of moxa smoke generation was equivalent to the cumulative amount of all substances contained in the sticks except for moisture, ash, and the oxygen consumed during combustion. As shown in Table 3, 1*g* moxa sticks produced 0.8446 times moxa smoke. Notably, the rate of smoke generation was required to calculate the concentration of moxa smoke in subsequent experiments.

### 3.2. Mortality and General Condition of Rats

Table 4 shows the results of acute toxicity tests used to assess the different groups of rats. The 14-day mortality rates of rats in groups A, B, C, D, and E were 100, 100, 66.7, 16.7, and 0%, respectively, which was consistent with the results of the preliminary experiment. The penises of dead rats in group C had a hard texture and showed white matter during autopsy. This matter was judged to be sperm. The mouths, front paws, and feet of the dead rats were open, and the mouths of the rats contained mucus, indicating that death was not caused by high temperature, external deficiency of yang, or disregard of the body. After exposure to moxa smoke, the behavior of the rats became inconsistent, and they showed increased activity and squinting of the eyes. At higher moxa smoke concentrations, the rats experienced shortness of breath, restlessness, and severe bleeding from the nasal region.

### 3.3. Organ Index of the Lungs

The lung indexes of group A (*P* < 0.05, *P* < 0.01) and group B (*P* < 0.05, *P* < 0.01) rats differed significantly from that of the control group rats, unlike the indexes of other groups (Table 5). The increased indexes of the experimental groups indicate that edema or lesions may have been present in the lungs of rats belonging to those groups.

### 3.4. LC_50_

The LC_50_ value of Qing moxa smoke was calculated as 537.65 g/m³. This value provides a basis for subsequent analysis of subchronic toxicity.

### 3.5. Microscopic Examination

**Figure 2:**
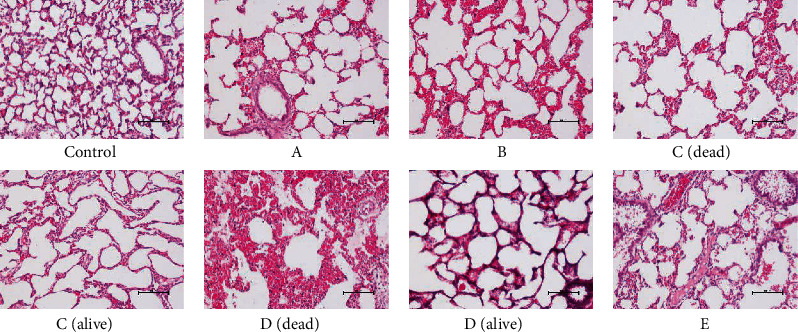
Microscopic images of lung pathology (H&E, ×200).

Microscopic observation of the lung tissues obtained from rats in different groups showed that exposure to moxa smoke increased inflammatory cell infiltration into the lungs (Figure 2). Moreover, the infiltration of inflammatory cells in the dead rats was greater than that observed in live rats. Group D rats, which died after 1 day of exposure, exhibited the most robust infiltration of inflammatory cells. The structure of alveolar cells in group E rats showed no obvious changes compared with those of control group rats; however, the alveolar structure showed changes at higher concentrations of moxa smoke.

### 3.6. RNA Sequencing: Quality Control, Assembly, and Mapping

High-throughput sequencing was used to systematically analyze gene expression in the transcriptome of lungs with or without exposure to moxa smoke. Our analysis of 24 samples yielded a total of 181.14 Gb clean reads, with more than 6.69 Gb clean reads for each sample. The percentage of the Q30 base was above 93.19%.  Table 6 summarizes the detailed mapping output obtained by mapping clean reads to the human reference genome. Alignment rates ranged between 93.9% and 95.47%, indicating high levels of gene expression in the four groups.

### 3.7. Functional Annotation and Gene Ontology Classification

Functional annotation provides information regarding protein function annotation, clusters of orthologous groups of proteins (COG) annotation, and gene ontology (GO) annotation. By aligning the transcripts and genes extracted from the lungs of our rats with publicly available protein databases such as KEGG, COG, and GO, 26,587 (97.14%) transcripts and 20,263 (96.26%) genes were successfully annotated (Table 7). Notably, most transcripts and genes were annotated using the COG database (94.79 and 94.90%, respectively), followed by GO (87.59 and 87.06%, respectively) and KEGG (67.54 and 64.86%, respectively).

### 3.8. Identification of Differentially Expressed Genes (DEGs)

**Figure 3:**
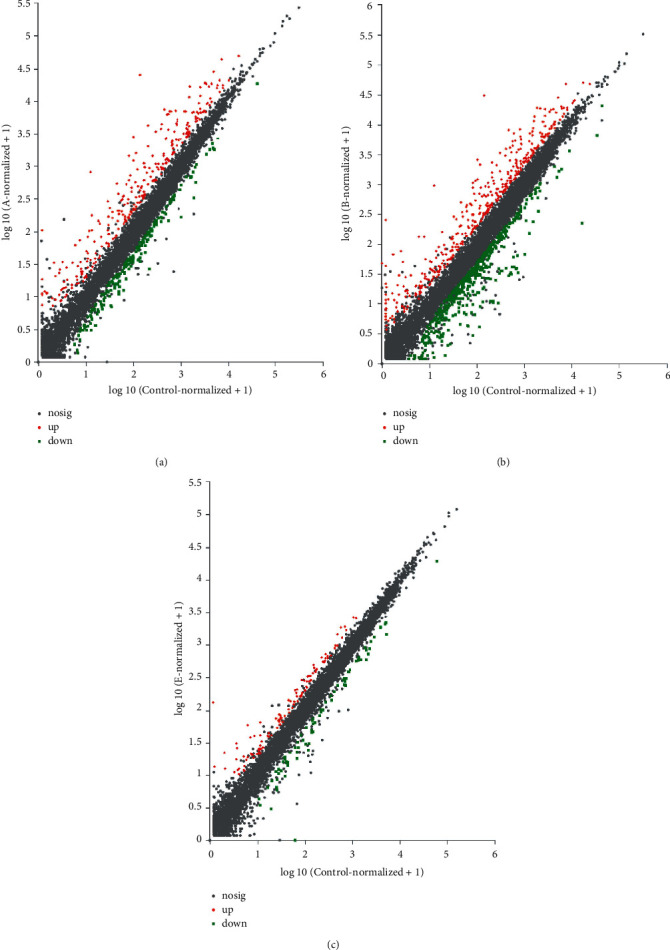
Scatter plots show differences in expression levels. The abscissa and ordinate represent the expression levels of genes/transcripts in the control and experimental groups, respectively. The values of horizontal and vertical coordinates were logarithmically transformed, and each point on the plot represents a specific gene/transcript. The abscissa value of a specific data point represents the expression level of a gene in the control sample, whereas the ordinate value represents the expression level of the same gene in the experimental sample. The red dots in the figure mark genes mark those showing significantly upregulated expression, whereas the blue dots mark those showing significantly downregulated expression. The gray dots indicate genes with no significant changes in expression levels. The greater the deviation from the diagonal line, the greater the difference in the expression level of a gene between the two samples; the closer a data point is to 0, the lower the expression level of that gene is.

Based on quantification of gene expression, the differentially expressed genes in groups A, B, and E and those in the control group were identified and compared using DESeq2 difference analysis software at the screening threshold of *P* < 0.05.  Figure 3 shows the scatter plots corresponding to the three groups. The expression levels of genes with log2FC fold change ≥1 were upregulated, whereas those of genes with log2FC fold change ≤ −1 were downregulated. Our results indicate that 286, 400, and 108 genes were upregulated, while 200, 477, and 78 genes were downregulated, in groups A, B, and E, respectively, compared with those of the control group (Table 8).

### 3.9. Gene Ontology Analysis of Differentially Expressed Genes

**Figure 4:**
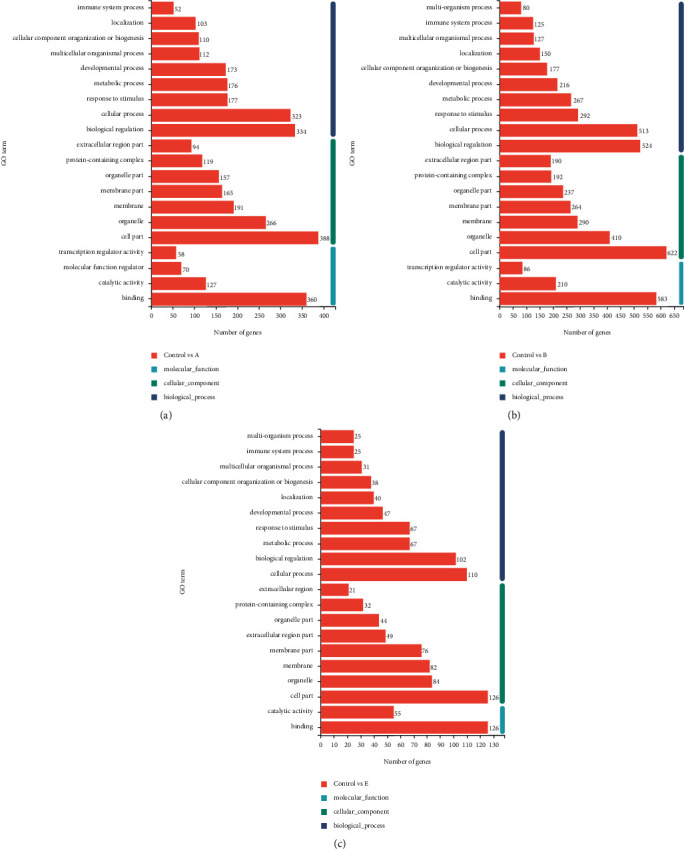
GO classification. The ordinate in the figure represents the secondary classification terms of GO. The upper abscissa represents the percentage of genes or transcripts contained in the secondary classification. The lower abscissa represents the number of genes/transcripts contained in the secondary classification. The three colors are used to indicate the three major categories.

To analyze the functional consequences of moxa smoke-induced changes in gene expression, we performed a GO enrichment analysis of DEGs based on the GO database. In terms of biological processes, our results showed that most DEGs in groups A and B were implicated in biological functions (334 and 524 genes, respectively), whereas most DEGs in group E were involved in cellular processes (110 genes) (Figure 4). In terms of cellular components, 388, 622, and 126 DEGs affected cellular components in groups A, B, and E, respectively. In groups A, B, and E, 360, 583, and 126 DEGs, respectively, were implicated in molecular functions. GO analysis demonstrated that cellular metabolism, cell communication, cell proliferation, and programmed cell death were the main biological processes.

### 3.10. KEGG Pathway Analysis of Differentially Expressed Genes

**Figure 5:**
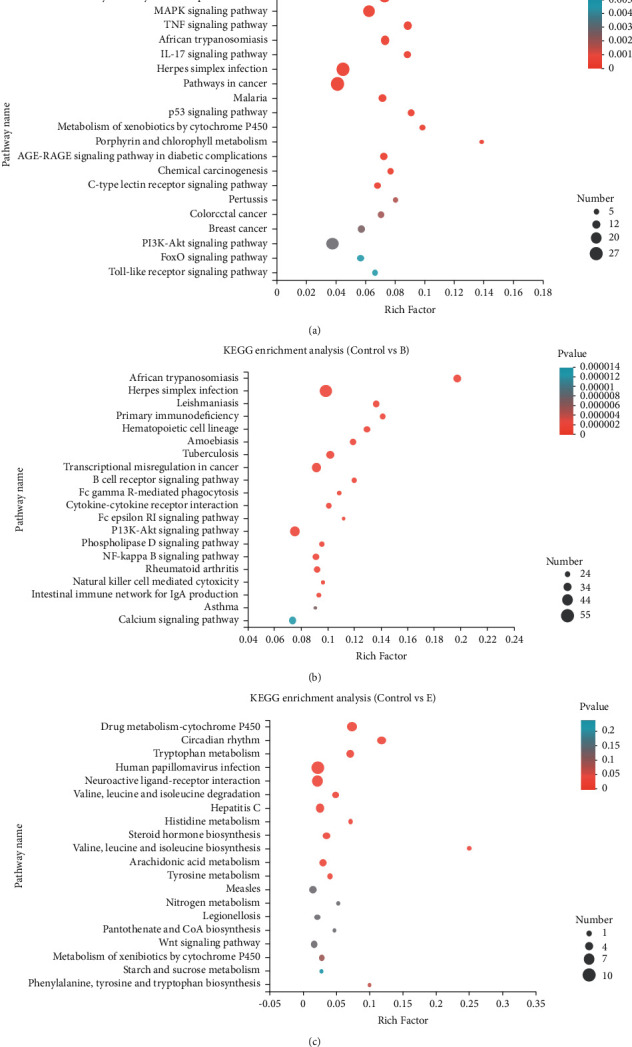
KEGG pathway enrichment analysis. The vertical axis shows the pathways; the horizontal axis represents the enrichment factor for a particular pathway. The size of the dot corresponds to the number of genes involved in the pathway. The color of the dot corresponds to the *P* value. Enrichment factor: the ratio of sample number to background number in the pathway.

In living organisms, different genes coordinate with each other to perform specific biological functions. Pathway analysis is an important tool that can be used to understand these functions. Our pathway functional significant enrichment analysis showed that DEGs were primarily associated with pathways involving biochemical metabolism and signal transduction. In groups A, B, and E, we detected 46, 57, and 12 pathways, respectively, showing significant differences in expression compared with those of the control group (*P* < 0.05).  Figure 5 depicts the top 20 enriched KEGG pathways based on RNA-seq data obtained in this study. These pathways were implicated in the development of cancer, PI3K-Akt signaling, and neuroactive ligand-receptor interaction, among others. Overall, our results provide a foundation for further analysis of the effects of moxa smoke on rats and for elucidation of the molecular mechanisms underlying these effects.

### 3.11. qRT-PCR Validation of Differentially Expressed Genes

**Figure 6:**
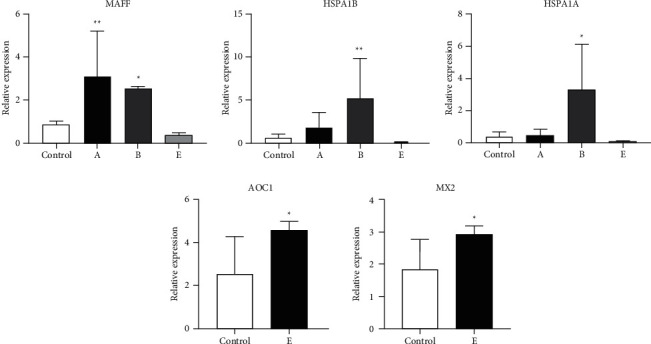
qRT-PCR analysis of the expression levels of the five DEGs examined in this study. Rats in groups A, B, and E were exposed to 151.33, 128.06, and 68.68 g of burned moxa sticks, respectively. Bars represent standard deviation (*n* = 6). Bars represent means ± SD. ^*∗*^*P* < 0.05; ^∗∗^*P* < 0.01.

To verify the credibility of our transcriptomic data, five DEGs, namely, *MAFF*, *HSPA1B*, *HSPA1A*, *AOC1*, and *MX2*, were selected for analysis using qRT-PCR. Our results confirmed that *MAFF*, *HSPA1B*, and *HSPA1A* were differentially expressed in all the experimental rats (dead and living) utilized in our present study; however, the expressions of these genes varied in different groups. The expression of DEGs *AOC1* and *MX2* was only detected in group E (all living rats).  Figure 6 shows the relative expression levels of these five genes in groups A, B, and E and in the control group. Overall, the expression levels obtained using qRT-PCR were consistent with those obtained using RNA-seq, indicating that RNA-seq data reliably reflected the changes in gene expression induced by exposure to moxa smoke. Results obtained using RNA-seq and qRT-PCR showed that the lungs of group A rats exhibited higher expression, whereas the lungs of group E rats exhibited lower expression of *MAFF*, *HSPA1B*, and *HSPA1A* compared with that of control group rats. Our results also indicate that the expression levels of *AOC1* and *MX2* increased after exposure to a minimal concentration of moxa smoke in rats from groups A, B, and C compared with the levels detected in control group rats.

## 4. Discussion

Based on the results of our acute toxicity assessment, two groups of patients can be clinically treated in a POAITANG moxibustion room (4 m long, 3 m wide, 3.6 m high; volume = 43200 L) within 2 h. Normally, a moxibustion room contains two beds, and 200 g moxa sticks are used to treat two patients simultaneously for 45 min. The poisoning cabinet (volume = 200 L) constructed in-house for our present study was much smaller than a clinical moxibustion room; however, in terms of Qing moxa sticks, the volume ratio of our emergency poisoning cabinet was comparable to that of a moxibustion room (216 : 1). That is, the amount of moxa sticks burned in a clinical setting for 2 h produces the same concentration of moxa smoke as 1 g of sticks burned in our poisoning cabinet. According to our results, the tolerable dose of moxa sticks was 68.68 times greater than the clinically used dosage. This value constitutes a reference for a safe dosage of moxa smoke in clinical moxibustion applications.

Previously, transcriptomic analysis was successfully used to identify DEGs in a mouse pulmonary poisoning model established using an intratracheal injection of ricin [22]. The results obtained in that study indicated that the expression levels of numerous genes related to leukocyte migration and chemotaxis increase continuously after exposure to ricin. The expression levels of genes involved in acute immune and inflammatory responses increase within 12 h of exposure to ricin. After only 4 h of exposure to ricin, 17 differentially expressed genes involved in ribotoxic stress response, endoplasmic reticulum stress response, and immune response in the lungs show increased expression levels. Although the effects of ricin on the expression of mouse genes have been well documented, the effects of moxa smoke on rat lungs and the mechanisms underlying these effects remain unclear because few studies have examined these factors. To the best of our knowledge, our present study is the first to report on the effects of moxa smoke on rat lungs assessed using acute toxicity testing. Herein, we used transcriptomic analysis to study changes in RNA expression levels in the lungs of rats exposed to varying concentrations of moxa smoke.

The KEGG pathway analysis performed in our present study demonstrated that the differentially expressed genes (DEGs) detected in rats exposed to high doses of moxa smoke (groups A and B) were primarily implicated in human disease pathways. The DEGs identified in group E rats exposed to a maximal tolerable dose of moxa smoke were mostly involved in metabolic pathways, especially in amino acid and lipid metabolism. The metabolism of amino acids affects protein synthesis and provides the functional groups needed to synthesize nucleic acids and fats, both of which participate in cellular metabolic processes [23–25]. Lipids are fundamental components of cell membranes and biofilms and are the main form of stored energy in most cells. Moreover, lipids regulate most signaling pathways in living organisms [26]. Therefore, the malfunctioning of lipid metabolism causes serious biological disorders [27].

*MAFF*, a member of the MAF family, is a transcription factor in the basic-leucine zipper that plays an important role in repressing gene expression by regulating the transcriptional repressors and activators using homodimers and heterodimers. Moreover, *MAFF* is involved in diverse physiological and pathological processes including hematopoiesis, cellular stress response, and cancer development [28–31]. According to previous studies, the antitumor effect of *MAFF* is regulated by miR-224-5p [32]. *HSPA1B* belongs to the HSP70 family whose expression level is known to increase rapidly during cell stress (such as heat shock) or viral infection. Genetic variations in HSP70 are involved in individual susceptibility to certain diseases via changes in protein expression or function. HSP70 can stabilize protein aggregation, mediate the folding of newly translated polypeptides in the cytoplasm and organelles, bind to nucleotides via an ATP-dependent process, and participate in stress response and cellular apoptosis [33]. As a polymorphic gene belonging to the HSP70 family, *HSPA1A* is commonly associated with a variety of disease symptoms and performs anti‐inflammatory and antiapoptotic functions [34]. *MAFF*, *HSPA1B*, and *HSPA1A* mainly act on pathways related to human diseases. The results obtained in our present study demonstrate that the expression levels of these genes in group E rats were lower than those in control group rats, indicating that the rats exposed to the maximal tolerable amount of moxa smoke did not exhibit a stress response. This result suggests that this amount may be utilized in clinical treatment using moxa smoke.

*AOC1* is a secreted diamine oxidase that is responsible for the degradation of highly expressed polyamines (such as putrescine and histamine) in the lungs, kidneys, placenta, and intestines [35]. In living organisms, the level of polyamines must fall within a very narrow range; otherwise, these compounds may cause serious physiological consequences, such as growth inhibition and apoptosis. Moreover, the metabolism of polyamines produces cytotoxic reactive oxygen species. These species promote cellular apoptosis by destroying cell membranes and regulating the permeability of mitochondrial membranes [36, 37]. Therefore, *AOC1* plays an important role in physiologic and pathologic processes by regulating polyamine homeostasis. *MX1* and *MX2*, two myxovirus resistance genes located on the human chromosome 21q22.3, encode the MXA and MXB proteins, respectively [38]. Sixty-three percent of the amino acid sequences of MXA and MXB are identical, and the three-dimensional structures of these two proteins are nearly overlapped. Recent studies have shown that MXB acts as an interferon-induced limiting factor that targets HIV-1 by binding to the HIV-1 capsid and inhibiting its nuclear import, leading to inhibition of replication in the postentry step [39–41]. This finding indicates that the MXB protein is involved in the innate immune response implicated in viral infection and tumor development. In addition, MXB contains a nuclear localization signal and appears in the nuclear pore complex, which suggests that it plays a crucial role in the cell cycle and affects nuclear import [42]. Overexpression of *MX2*, the gene encoding MXB, significantly inhibits cell proliferation, which suggests that it may prolong the cell cycle of glioma cells by inhibiting their invasiveness and migratory capacity. Hence, *MX2* is a potential biomarker that may be used to diagnose glioblastoma [43]. Transcriptomic sequencing and qRT-PCR results obtained in our present study showed that the rats in group E exhibited increased expression levels of *AOC1* and *MX2*. Therefore, exposure to the maximal tolerable dose of moxa smoke may promote growth and development in rats.

Overall, our results demonstrate that the toxicity of moxa smoke was closely related to dosage and that the dosage normally used in clinical moxibustion rooms poses no health risks. High doses of moxa smoke displayed a significant suppression effect on the function of rat lungs, which was a result of interactions among multiple pathways and signal molecules. Interfering a series of human diseases pathways including legionellosis, toxoplasmosis, and measles and some key factors such as *MAFF*, *HSPA1A*, *HSPA1B*, *AOC1,* and *MX2*, high doses of moxa smoke influenced the function of rat lungs. In addition to this important finding, we also designed a novel acute poisoning cabinet that can be used as a valuable tool in future studies assessing moxa smoke toxicity. The innovative moxa smoke absorption and filtration device, composed of five solvents having different polarities, is also useful because it can effectively capture all components in the smoke while maintaining a dynamic balance of gases in the poisoning cabinet. In summary, our findings indicate that moxibustion is relatively safe in the clinical setting; however, further safety assessment of moxa smoke is needed and will be investigated in future studies.

## Figures and Tables

**Table 1:** Moisture content of moxa sticks.

No.	Initial weight (g)	Final weight (g)	Absolute moisture content (%)	Relative moisture content (%)
1	3.03	2.73	10.99	9.90
2	3.56	3.25	9.54	8.71
3	3.25	2.95	10.17	9.23
4	3.48	3.15	10.48	9.48
5	3.42	3.09	10.68	9.65
6	3.12	2.79	11.83	10.58
Mean ± SD	3.31 ± 0.21	2.99 ± 0.21	10.62 ± 0.77	9.59 ± 0.63

**Table 2:** Ash content of moxa sticks.

No.	Weight of moxa stick (g)	Weight of ash (g)	Absolute ash content (%)	Relative ash content (%)
1	3.46	0.25	7.99	7.23
2	3.37	0.24	7.88	7.12
3	3.69	0.26	7.79	7.05
4	3.52	0.25	7.86	7.10
5	3.35	0.24	7.92	7.16
6	3.53	0.25	7.83	7.08
Mean ± SD	3.49 ± 0.12	0.25 ± 0.01	7.88 ± 0.07	7.12 ± 0.06

**Table 3:** The rate of moxa smoke generation.

No.	Weight of moxa stick (g)	O_2_ Concentration before combustion (%)	O_2_ Concentration after combustion (%)	Weight of ash (g)	Rate of moxa smoke generation (%)
1	10.66	21.4	20.9	0.76	84.62
2	24.32	20.9	20.0	1.73	84.33
3	39.94	21.4	19.8	2.84	84.43
Mean ± SD	24.97 ± 14.65	21.23 ± 0.29	20.23 ± 0.59	1.78 ± 1.04	84.46 ± 0.15

**Table 4:** Results of acute toxicity assessment.

Group	Animal number	Body weight (*x*x(−) ± *s*)	Weight of moxa stick (g)	Dose (g/m^3^)	Number of dead rats	Mortality rate (*P*)	*P* ^2^
A	6	307.62 ± 7.05	151.33	639.067	6	100%	1
B	6	318.97 ± 16.62	128.06	540.797	6	100%	1
C	6	361.99 ± 9.75	112.51	475.130	4	66.7%	0.445
D	6	310.51 ± 8.38	86.41	364.909	1	16.7%	0.028
E	6	339.67 ± 9.97	68.68	290.036	0	0	0

**Table 5:** Organ index.

Group	Animal number	Number of moxa stick	Weight of moxa stick (g)	Lung weight/body weight (mg/g)
Control	6	—	—	4.93 ± 0.57
A	6	7	151.33	7.67 ± 1.56^∗∗^
B	6	6	128.06	7.17 ± 1.68^∗∗^
C	6	5	112.51	5.58 ± 1.21
D	6	4	86.41	6.40 ± 2.29
E	6	3	68.68	5.23 ± 0.51

**Table 6:** Summary of trimming and read mapping of RNA sequences extracted from lung tissues with or without exposure to moxa smoke.

Sample	Raw reads	Clean reads	Total mapped	Multiple mapped	Uniquely mapped
Control-1	50,173,870	49,796,126	47,428,880 (95.25%)	2,917,603 (5.86%)	44,511,277 (89.39%)
Control-2	46,114,692	45,781,086	42,990,282 (93.9%)	2,799,797 (6.12%)	40,190,485 (87.79%)
Control-3	52,445,922	52,079,738	49,504,389 (95.05%)	3,096,088 (5.94%)	46,408,301 (89.11%)
Control-4	48,545,612	48,217,204	45,990,652 (95.38%)	2,991,351 (6.2%)	42,999,301 (89.18%)
Control-5	50,992,444	50,562,024	48,161,499 (95.25%)	3,224,105 (6.38%)	44,937,394 (88.88%)
Control-6	50,544,782	50,169,430	47,895,334 (95.47%)	3,106,564 (6.19%)	44,788,770 (89.28%)
A-1	44,935,518	44,575,026	42,197,588 (94.67%)	3,049,901 (6.84%)	39,147,687 (87.82%)
A-2	50,700,004	50,338,410	47,993,769 (95.34%)	3,186,028 (6.33%)	44,807,741 (89.01%)
A-3	50,236,082	49,848,242	47,350,398 (94.99%)	3,206,303 (6.43%)	44,144,095 (88.56%)
A-4	48,693,504	48,333,432	45,959,510 (95.09%)	2,982,521 (6.17%)	42,976,989 (88.92%)
A-5	49,146,808	48,759,042	46,340,404 (95.04%)	2,945,158 (6.04%)	43,395,246 (89.0%)
A-6	53,718,716	53,311,266	50,690,875 (95.08%)	3,264,160 (6.12%)	47,426,715 (88.96%)
B-1	55,820,604	55,423,436	52,745,165 (95.17%)	3,614,618 (6.52%)	49,130,547 (88.65%)
B-2	53,589,380	53,111,498	50,506,434 (95.1%)	3,334,875 (6.28%)	47,171,559 (88.82%)
B-3	55,490,490	55,043,366	52,450,686 (95.29%)	3,366,022 (6.12%)	49,084,664 (89.17%)
B-4	49,064,936	48,628,478	46,071,906 (94.74%)	3,156,726 (6.49%)	42,915,180 (88.25%)
B-5	54,094,302	53,605,834	50,848,156 (94.86%)	3,618,857 (6.75%)	47,229,299 (88.1%)
B-6	50,670,142	50,301,200	47,808,555 (95.04%)	3,146,533 (6.26%)	44,662,022 (88.79%)
E-1	48,835,796	48,411,848	46,031,928 (95.08%)	3,079,255 (6.36%)	42,952,673 (88.72%)
E-2	48,866,976	48,537,232	46,117,663 (95.02%)	3,002,555 (6.19%)	43,115,108 (88.83%)
E-3	55,030,908	54,608,348	51,926,672 (95.09%)	3,335,432 (6.11%)	48,591,240 (88.98%)
E-4	51,144,428	50,682,046	48,198,708 (95.1%)	3,001,808 (5.92%)	45,196,900 (89.18%)
E-5	46,247,988	45,888,214	43,493,626 (94.78%)	2,855,318 (6.22%)	40,638,308 (88.56%)
E-6	52,636,034	52,255,560	49,672,774 (95.06%)	3,101,746 (5.94%)	46,571,028 (89.12%)

*Note.* (1) Sample: sample name. (2) Total reads: the number of filtered sequences (clean reads). (3) Total mapped: the number of clean reads that can be mapped to the genome. (4) Multiple mapped: the number of clean reads with multiple alignment positions on the reference sequence. (5) Unique mapped: clean reads with unique alignment position on the reference sequence.

**Table 7:** Functional annotation of transcriptome data available in three publicly available protein databases.

Type	Transcript number (percentage)	Gene number (percent)
KEGG	18484 (67.54%)	13653 (64.86%)
GO	23972 (87.59%)	18327 (87.06%)
COG	25944 (94.79%)	19765 (93.90%)
Total annotation	26587 (97.14%)	20263 (96.26%)
Total	27369	21050

**Table 8:** Number of differentially expressed genes (DEGs) based on the criteria of |log2FC| ≥ 1 and *P* < 0.05.

Group	Total DEG	Upregulated	Downregulated
Control vs. A	486	286	200
Control vs. B	877	400	477
Control vs. E	186	108	78

## Data Availability

The data used to support the findings obtained in this study are available from the first author upon request.
